# Gene expression meta-analysis of Parkinson’s disease and its relationship with Alzheimer’s disease

**DOI:** 10.1186/s13041-019-0436-5

**Published:** 2019-02-28

**Authors:** Jack Kelly, Rana Moyeed, Camille Carroll, Diego Albani, Xinzhong Li

**Affiliations:** 1 Faculty of Medicine and Dentistry, Plymouth University, Plymouth, PL6 8BU UK; 2 Faculty of Science and Engineering, Plymouth University, Plymouth, PL6 8BU UK; 3 Department of Neuroscience, IRCCS - Istituto di Ricerche Farmacologiche “Mario Negri” Via La Masa 19, 20156 Milan, Italy; 4 School of Science, Engineering & Design, Teesside University, Middlesbrough, TS1 3BX UK

**Keywords:** Systems analysis, Parkinson’s disease, Alzheimer’s disease, Meta-analysis, Gene expression, Transcriptome analysis

## Abstract

Parkinson’s disease (PD) and Alzheimer’s disease (AD) are the most common neurodegenerative diseases and have been suggested to share common pathological and physiological links. Understanding the cross-talk between them could reveal potentials for the development of new strategies for early diagnosis and therapeutic intervention thus improving the quality of life of those affected. Here we have conducted a novel meta-analysis to identify differentially expressed genes (DEGs) in PD microarray datasets comprising 69 PD and 57 control brain samples which is the biggest cohort for such studies to date. Using identified DEGs, we performed pathway, upstream and protein-protein interaction analysis. We identified 1046 DEGs, of which a majority (739/1046) were downregulated in PD. *YWHAZ* and other genes coding 14–3-3 proteins are identified as important DEGs in signaling pathways and in protein-protein interaction networks (PPIN). Perturbed pathways also include mitochondrial dysfunction and oxidative stress. There was a significant overlap in DEGs between PD and AD, and over 99% of these were differentially expressed in the same up or down direction across the diseases. *REST* was identified as an upstream regulator in both diseases. Our study demonstrates that PD and AD share significant common DEGs and pathways, and identifies novel genes, pathways and upstream regulators which may be important targets for therapy in both diseases.

**Electronic supplementary material:**

The online version of this article (10.1186/s13041-019-0436-5) contains supplementary material, which is available to authorized users.

## Introduction

Parkinson’s disease (PD) is the second most prevalent neurodegenerative disease (ND) effecting approximately 145,000 people in the UK [1]. With an ageing population, it is predicted that the number of PD patients in the UK will increase by 18.1% between 2015 and 2065 [1]. In the US it is predicted that PD cases will increase from 680,000 to 1,238,000 by 2030 [2]. PD primarily affects motor systems of the central nervous system because of the death of dopamine generating cells in the substantia nigra (SN) in the midbrain [3]. The main neuropathologic hallmark of PD is the accumulation of α-synuclein in neurons in the form of Lewy bodies [3].

Alzheimer’s disease (AD) is the most common ND accounting for 60–80% of dementia cases, characterised pathologically by deposits of intracellular tau neurofibrillary tangles and accumulation of extracellular amyloid β (Aβ) plaques in the brain [4]. The most common clinical symptom of AD is gradual progressive memory loss that eventually affects other cognitive functions such as communication and movement. There are currently many promising advances in the understanding of AD, including novel biomarkers [5, 6] and underlying biological mechanisms [7].

There is increasing evidence that PD and AD both have several common characteristics [8]. Around 80% of PD patients develop dementia over time, with the average time from onset of PD to dementia being 10 years [9]. PD and AD are both age-related diseases that have hallmarks of protein aggregation, indeed α-synuclein is found as a non-amyloid component within AD amyloid plaques and over 60% of AD cases are accompanied by the formation of Lewy bodies [10]. There are certain genetic variants that increase both PD and AD risk, for example the strong risk factor for AD, *APOE4*, has been shown to be related to cognitive decline in PD [11]. There is evidence that molecular pathways, including mitochondrial function, oxidative stress and inflammation underlie the pathogenesis of both AD and PD, however, the pathogenic mechanisms of both diseases have not been entirely explained [8]. There has been found a co-occurrence of Aβ, tau and α-synuclein pathology within neurons and oligodendrocytes from post-mortem brain tissue derived from those with AD and PD [12]. Complex interactions between these proteins can seed the aggregation of each another, though the underlying cause of this is not yet understood [12].

The largest RNA sequencing (RNA-seq) study in the PD brain was performed using prefrontal cortex tissue, and subset of these samples were tested using proteomics [13]. This study gives excellent insight into the transcriptomic and proteomic changes that occur within the frontal cortex of PD patients highlighting disruptions in protein folding, mitochondrial pathways and ubiquitin conjugation pathway, reflecting processes that are characteristic of PD. However, as the prefrontal cortex is not the primary brain region effected in PD, in some cases the PD could have had a minimal effect [14].

A recent review has highlighted the previous transcriptomics studies published about PD [15]. This review highlights the limitation of small samples sizes in many transcriptomic studies of PD even when not restricted to the SN, demonstrating the need for meta-analysis to increase the power of these previous studies. In addition, it has been shown that there are low similarities between results of previous PD microarray studies in both human and animal tissues, due to the small sample sizes and differing microarray platforms used across studies [16]. Use of meta-analysis methods to increase the statistical power of studies as a result of increasing sample size has been successful in the past in identifying PGC-1α as a potential therapeutic target in PD [17]. Other previous brain microarray meta-analyses have used data from all brain regions available, ignoring region differences in the brain. Making the data used independent to a brain region is important as processes involved in PD can occur dependent on region. However, several previous meta-analysis studies have included repeated samples from patients being analysed using multiple different platforms [18] or multiple areas of the SN being analysed in the same patients. Including these, as previous meta-analyses have done [19–21], may introduce bias of results towards these individuals.

In this study we carry out an integrated study to give insight into the genomics, genetics and molecular mechanisms that underlie the features of PD, and reveal the relationship with AD. Here we apply a novel meta-analysis approach we proposed [7] to discover differentially expressed genes (DEGs) in PD and then make comparison to AD. The data of the SN was chosen for this meta-analysis as degeneration of neurons in the SN is a hallmark of the disease [8] and has the largest amount of microarray data available. Our meta-analysis approach avoids relying exclusively on the genes that have expression data for each constituent study, as previous PD SN meta-analysis have done [22, 23], therefore may lead to novel discovery.

## Materials and methods

### Data collection and pre-processing

We searched arrayExpress (https://www.ebi.ac.uk/arrayexpress/) and NCBI GEO (Gene Expression Omnibus) (http://www.ncbi.nlm.nih.gov/geo/) databases using the keywords “Parkinson AND substantia nigra” to find mRNA expression studies of human post-mortem brain tissue from the SN related to PD. Studies were included if they: (1) used clinically diagnosed idiopathic PD patients; (2) used brain tissue samples and (3) had cohorts with more than three samples in either disease or control conditions. If a patient had duplicate samples analysed using different platforms or multiple samples from within the SN, only one of them was used.

**Fig. 1:**
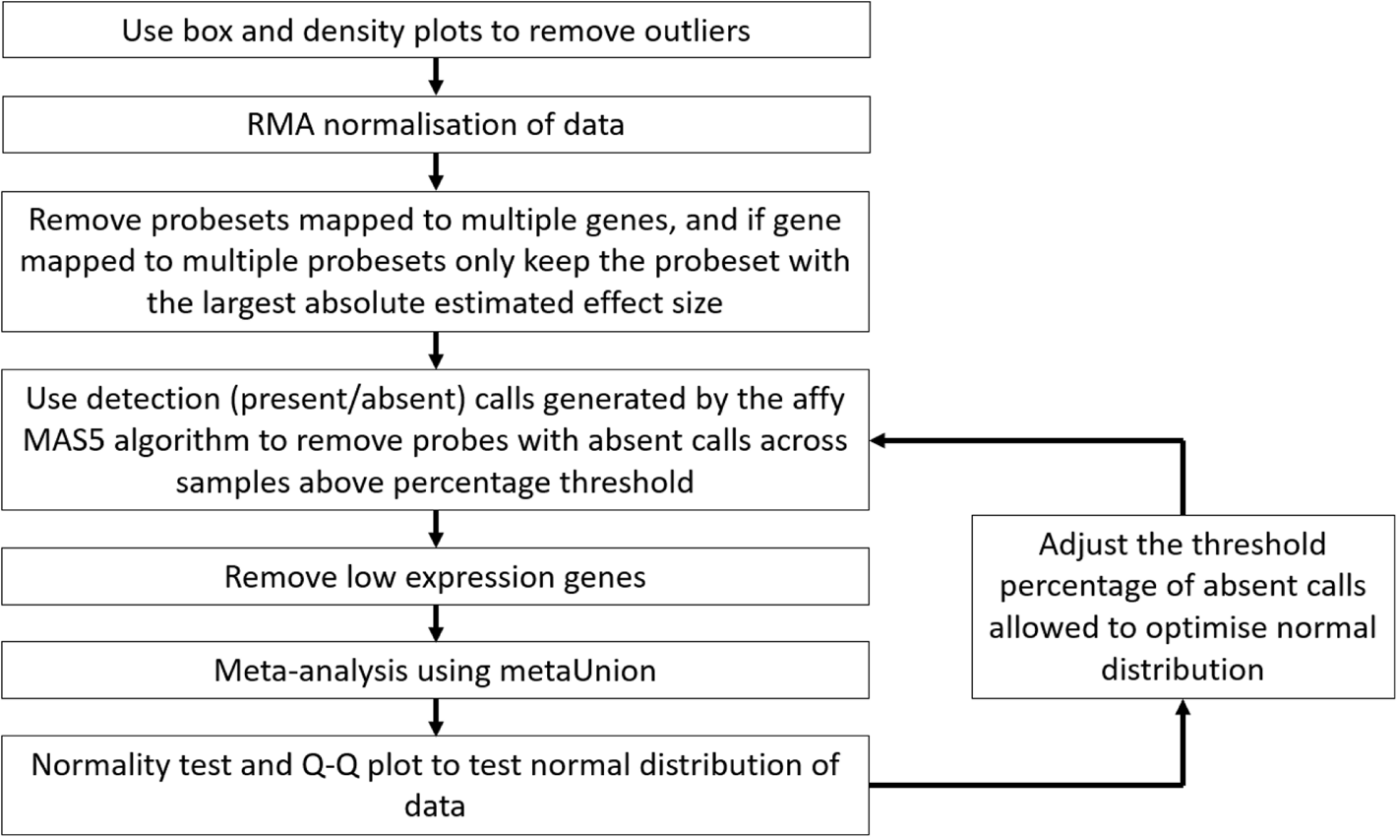
Workflow of data processing. Outlier samples were removed, and data normalized before the detection (Present/Absent) call algorithm was used to remove data that was not reliably detected. For each study, probesets with absent calls across a chosen percentage of samples were removed. This was repeated in 5% intervals removing probesets with 5% up to 95% of samples absent. The percentage absent cut-off used was set to optimize the normal distribution of the data. After this, the bottom 5% of average expression values across samples was removed and meta-analysis performed

Data processing is shown in Fig. 1. All work was done in the R programming language [24]. The identified datasets were downloaded and raw CEL file data were loaded into R using the affy package available on bioconductor (http://www.bioconductor.org) [25]. Boxplots and density plots were used to identify any outlier samples that were subsequently removed. The datasets were then normalized using the Robust Multi-array Average (RMA) approach in the affy R package. Probesets were first mapped to Entrez Gene IDs using manufacturer-supplied annotation files. Probesets that mapped to multiple genes were removed, and for any genes that mapped to multiple probesets only the probeset that had the largest absolute estimated effect size was kept [7].

The first step of pre-filtering was using detection (Present/Absent) call generated by the affy microarray suite version 5 (MAS5) algorithm to remove data that was not reliably detected. For each study, probesets with absent calls across a chosen percentage of samples were removed. This was repeated in 5% intervals removing probesets with 5% up to 95% of samples absent. The percentage absent cut-off used was set to minimize the *p*-value of the Anderson-Darling normality test using the nortest R package [26] and give optimum Quantile-Quantile (Q-Q) plots of the meta-analysis z-score results. This was done to reduce how arbitrary the selected filtering parameters are. After this, the bottom 5% of average expression values across samples was removed to reduce low expression data noise.

The Genotype-Tissue Expression (GTEx) database [27] contains RNA-seq data for SN tissue which were used to test robustness of our control data. The RNA-seq Gene transcripts per kilobase million (TPM) from GTEx analysis v7 were downloaded (available at https://gtexportal.org/home/datasets). Genes that mapped to more than one gene symbol and any duplicated gene symbols were removed. All RMA normalized microarray control data were merged using the ComBat function [28] from the sva R package [29]. The Pearson correlation coefficient between the average expression levels for the microarray and the average log2 TPM of the RNA-seq was then calculated.

### Meta-analysis

Meta-analysis was performed using the novel metaUnion R package previously proposed by us [7] (available at https://github.com/chingtoe365/metaUnion). This meta-analysis method calculates the combined effect size across studies to identify DEGs with the assumption of a normal distribution of the data. Our approach works on the combined gene sets from all the studies included in the meta-analysis, rather than the genes that are common between all datasets as other approaches have done [22, 23]. The metaUnion package is adapted to include age and gender as covariates in the model, implemented using limma [30].

### Identification of activated transcriptional regulators, pathway analysis and protein-protein interaction network analysis

The QIAGEN Ingenuity® Pathway Analysis (IPA®, QIAGEN Redwood City, www.qiagen.com/ingenuity) software was used to analyse canonical pathways and upstream regulator analysis (URA) [31] of the DEGs. The canonical pathways with Benjamini-Hochberg corrected *p*-values below 0.05 and upstream regulators with *p*-values below 0.01 are considered significant.

A protein-protein interaction network (PPIN) is used to analyse the interaction of DEGs at the protein level. The PPIN from the Human Protein Reference Database (HPRD, release 9) is downloaded and visualized in Cytoscape v.3.6.1 [32] to create a whole human PPIN with 9617 unique protein entries (nodes) and 39,240 unique undirected interactions (edges). We then mapped the DEGs and known risk loci for PD identified by a recent GWAS meta-analysis to build a subnetwork [33].

### Comparison to Alzheimer’s data

Our results were compared to our previous study using similar methodology on AD frontal cortex microarray data [7]. The significance of the DEGs shared between AD and PD was determined using a two-tailed Fisher’s exact test and DEGs in common are tested for significant distribution up or down regulation using a Sign test. We identified pathways perturbed in both PD and AD in addition to those unique to each disease. In addition, pathway analysis was done on DEGs unique to each disease and DEGs shared between diseases.

## Results

### Data sets collected for this study

Our search criteria found 7 Affymetrix chip datasets which included 69 PD and 57 control samples. Information about the datasets is shown in Additional file 1: Table S1. After several rounds of calculation with different filtering threshold (see methods), we identified the optimal detection call threshold of 15% absent to give data closest to normal distribution (shown in Additional file 1: Figure S1).

### Meta-analysis

Meta-analysis identified 1046 DEGs from the initial pool of 10,362 genes after false discovery rate (FDR) correction (FDR *p*-value < 0.05), of which 307 were upregulated and 739 were downregulated. A full list of the 1046 DEGs are shown in Additional file 2 (also available at https://figshare.com/s/508c83677f885ced28dc). Table 1 lists the top 30 most significant DEGs, sorted by FDR adjusted *p*-value, of which only three are up-regulated.

**Table 1:** Top 30 most significant differentially expressed genes found in out meta-analysis

Gene name	Entrez ID	Average FC^a^	metaZscore	Effect^b^	FDR corrected Pval
YWHAZ	7534	0.52	−6.26	–	4.09E-06
SNCA	6622	0.57	− 6.00	–	1.03E-05
DCLK1	9201	0.52	−5.91	–	1.08E-05
GBE1	2632	0.43	−5.88	-?-----	1.08E-05
PAIP1	10,605	0.53	−5.61	------?	4.06E-05
TMEM255A	55,026	0.39	−5.58	-??---?	4.06E-05
OLFM1	10,439	0.48	−5.33	--?---?	1.31E-04
OPA1	4976	0.59	−5.32	------?	1.31E-04
HPRT1	3251	0.45	−5.30	–	1.31E-04
PPP3CB	5532	0.54	− 5.25	–	1.41E-04
PDXK	8566	0.67	−5.24	–	1.41E-04
SLC18A2	6571	0.31	−5.24	-?-?---	1.41E-04
MDH2	4191	0.60	−5.21	–	1.50E-04
CHN1	1123	0.54	− 5.17	–	1.77E-04
RAB2A	5862	0.62	−5.10	–	2.37E-04
RUFY1	80,230	1.27	5.04	++?+++?	3.01E-04
CDH8	1006	0.47	−5.00	-????-?	3.47E-04
UBE2N	7334	0.66	−4.93	–	4.55E-04
ENSA	2029	0.67	−4.93	–	4.55E-04
SERINC3	10,955	0.63	− 4.89	–	4.86E-04
FGF13	2258	0.41	−4.88	–	4.86E-04
ATP6V1D	51,382	0.57	−4.87	–	4.86E-04
FRRS1L	23,732	0.54	−4.87	--?---?	4.86E-04
CDK14	5218	0.67	−4.86	--?----	4.86E-04
LHPP	64,077	1.43	4.86	++?++++	4.86E-04
AASDHPPT	60,496	0.60	−4.81	–	5.97E-04
SH3BP4	23,677	1.34	4.80	++?+++−	6.08E-04
REEP1	65,055	0.45	−4.75	--?---?	7.41E-04
FBXO9	26,268	0.65	−4.74	------?	7.47E-04
APLP2	334	0.72	−4.72	–	8.04E-04

^a^Average Fold Change

^b^”+/−/?” indicates up/down and missing in each individual study

A recent meta-analysis of GWAS data identified 69 risk genes for PD [33] only 49 of which were present in our initial gene pool and 9 were identified as DEGs, including *SNCA*, *ANK2* and *MAPT* (shown in Additional file 1: Table S2). We found that DEGs were more likely to contain disease associated variants than non-DEGs, however the significance of this is not very strong (OR = 2.25, 95% CI 0.96 ~ 4.72, *p*-value = 0.041, Fisher Exact test).

### Identification of activated transcriptional regulator, pathway analysis and protein-protein interaction network analysis

**Fig. 2:**
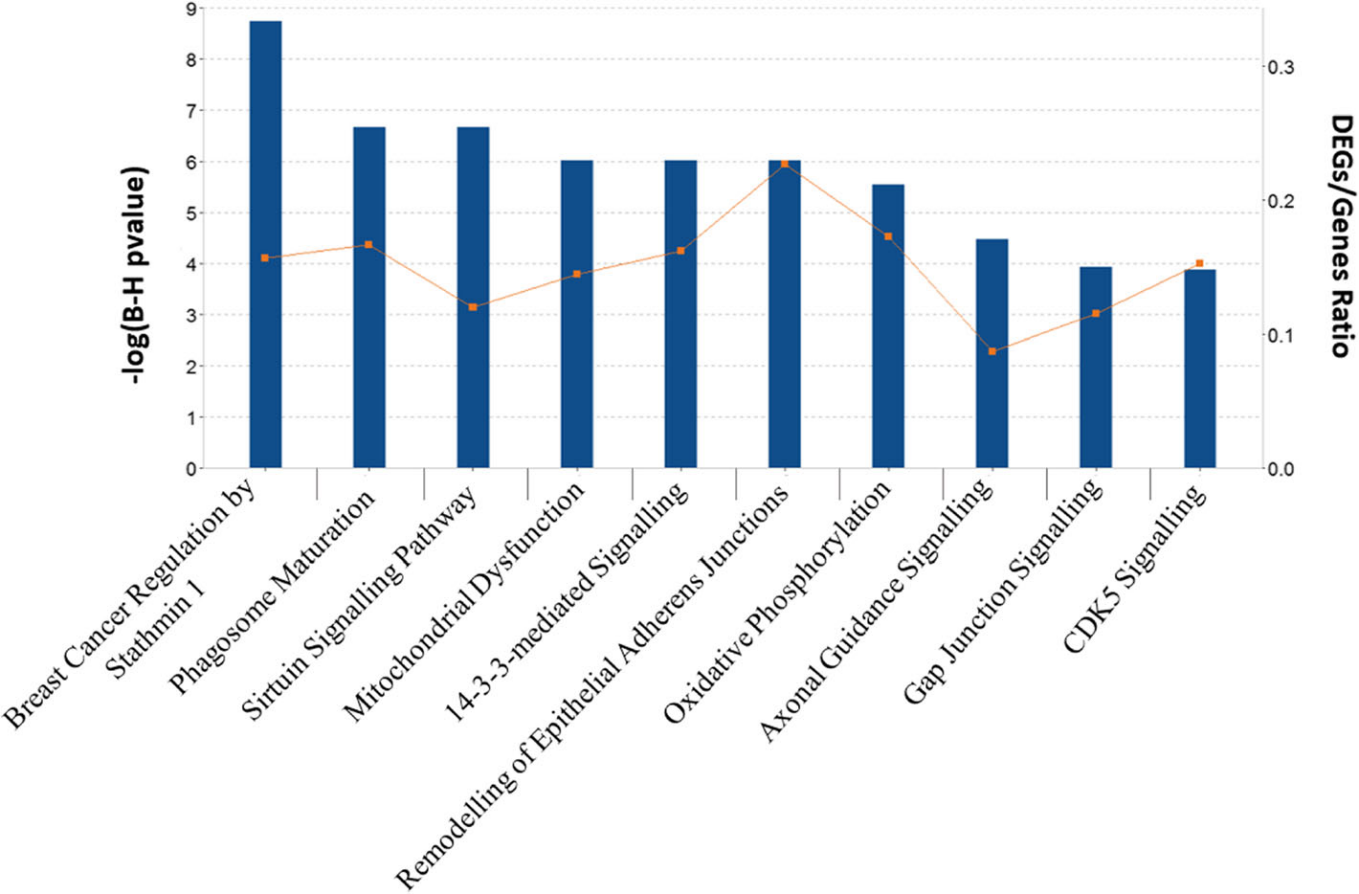
Top 10 most significant pathways identified using the downregulated DEGs

After Benjamini-Hochberg correction IPA identified 54 canonical pathways that were significant for the 1046 DEGs (Additional file 1: Table S3). Pathways identified include Sirtuin Signalling pathway (adjusted *p*-value = 2.18E-07, ratio = 34/283) and 14–3-3 mediated Signalling (adjusted *p*-value = 9.56E-07, ratio = 21/130). Using the downregulated DEGs 81 significant pathways were found (Additional file 1: Table S4). The top ten pathways identified by the downregulated DEGs are shown in Fig. 2. Using the upregulated DEGs, no significantly perturbed pathways were identified by applying multiple testing. Using less stringent nominal *p*-value, ten pathways were identified (*p*-value< 0.01), including Adipogenesis pathway (*p*-value = 2.04E-04, ratio = 9/132) and STAT3 pathway (*p*-value = 7.41E-04, ratio = 7/97). Using down-regulated DEGs IPA identified 17 upstream regulators (Additional file 1: Table S5) including transcription factor (TF) *REST* (*p*-value = 2.91E-04), which regulates six down regulated genes (*GAP43, INA, SCG2, SNAP25, TUBB3, UCHL1*). Using up-regulated DEGs IPA identified 25 upstream regulators including HSF1 (*p*-value = 1.57E-04) which regulates 8 upregulated DEGs.

**Fig. 3:**
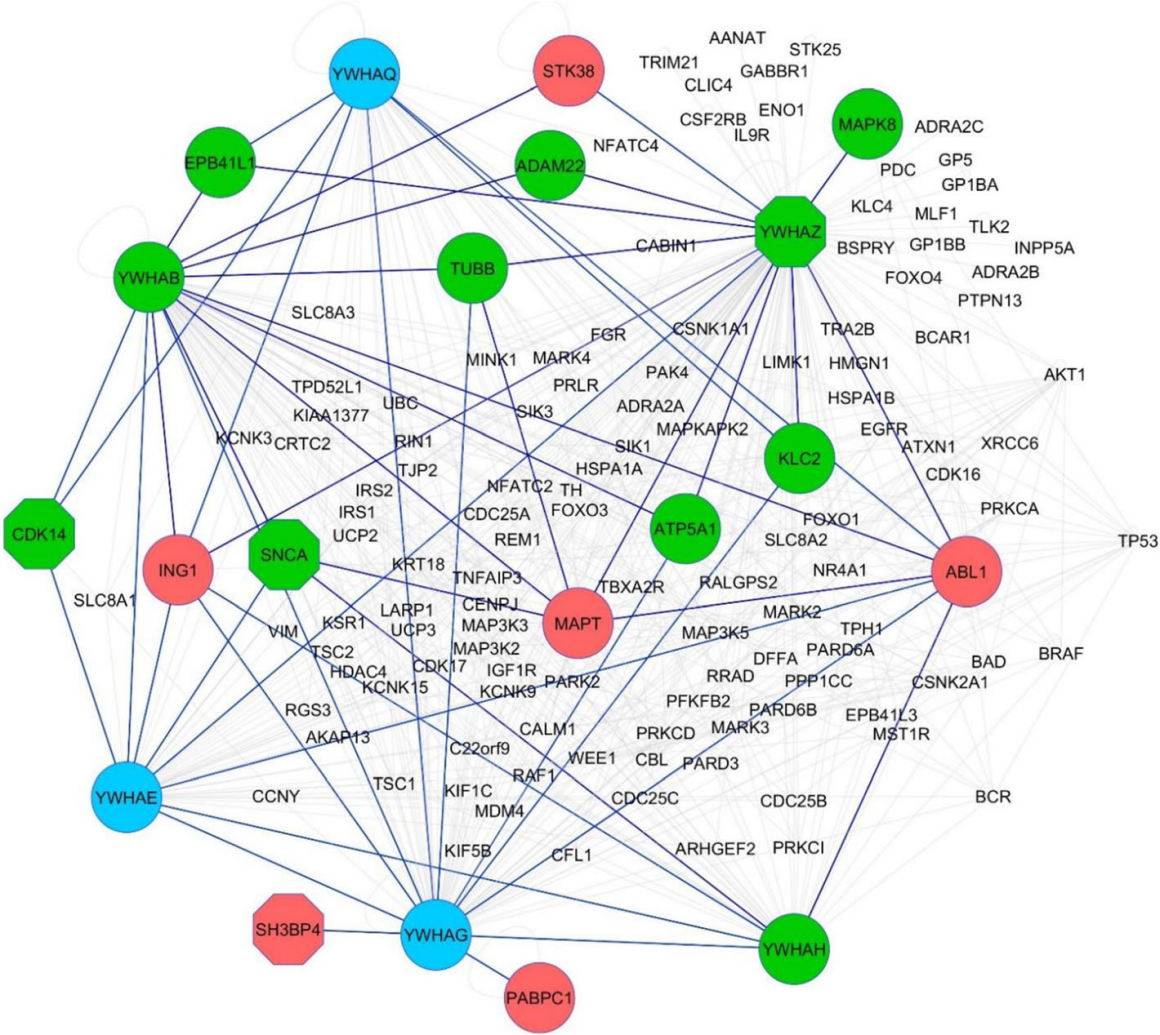
Protein-protein interaction subnetwork created using the first neighbour nodes of the 14–3-3 protein family in the DEG PPIN. Six 14–3-3 family genes, YWHAZ, YWHAB, YWHAG, YWHAE, YWHAQ and YWHAH, were in the top 10 hubs for the subnetwork created from the top 30 DEGs found in our PD meta-analysis. A subnetwork of these 14–3-3 family members and their first neighbours were created. There were 18 DEGs that mapped to this, with red nodes indicating upregulated genes and green nodes indicating downregulated genes. Blue nodes indicate 14–3-3 family members that are not PD DEGs. Octagons denote genes that were in the top 30 DEGs. This first neighbour network contains 139 nodes and 539 edges

A PPIN was created to understand relationships among top DEGs at a protein level. From the top 30 DEGs, 21 were mapped to the PPIN and first neighbour nodes (FNN) extracted. This subnetwork contains 248 nodes and 912 edges, and included 2 GWAS genes, *SNCA* and *MAPT*. The top 10 hubs, which have the greatest number of first neighbour connections, are shown in Additional file 1: Table S6. Of the top ten hubs, 6 belonged to the 14–3-3 family of proteins, including 14–3-3 zeta (*YWHAZ*) which is connected to 122 other genes in the subnetwork including 6 down and 4 upregulated DEGs. Figure 3 shows a subnetwork created using the FNN of the 14–3-3 protein family in the top 30 DEG PPIN.

Of the 69 GWAS genes previously identified, 37 mapped to the PPIN created. The subnetwork created had 331 nodes and 1245 edges that included 45 DEGs, including *SNCA*, *YWHAZ* and *MAPT*. DEGs were over-represented in the GWAS PPI sub-network (hypergeometric test, *p*-value = 1.05E-06). The largest hub of the GWAS gene PPI subnetwork was *MAPT* which had 46 mapped genes, followed by *DLG4* and *SNCA*.

### Comparison to Alzheimer’s disease

The PD DEGs identified in this study were compared to the 3124 AD DEGs previously found [7]. Between PD and AD, there were 436 DEGs in common (shown in Additional file 2), an overlapping analysis showed that is not just a chance event (OR = 4.32, 95%CI 3.79 ~ 4.93, *p*-value = < 2.2e-16, Fisher Exact test). This means around 42% of PD DEGs were found in AD and around 14% of AD DEGs were found in PD. Over 99% (432) of the shared DEGs were differentially expressed in the same up or down direction. *PIK3R3, LIMK2, CD55* and *MAPT* were the only genes not dysregulated in the same direction between diseases. It is interesting that the majority of DEGs in common between AD and PD were significantly distributed towards downregulation (two-tailed sign test *p*-value < 2.2E-16) (see Additional file 1: Table S7).

IPA identified 54 affected pathways in PD and 107 pathways in AD, with 27 shared between these two (Additional file 1: Table S3). Interestingly, many of the top pathways in PD were also dysregulated in AD, including Sirtuin Signalling pathway (AD adjusted *p*-value = 3.39E-04) and 14–3-3-mediated Signalling (AD adjusted *p*-value = 5.13E-03). The top five pathways identified using DEGs unique to PD were all among the common pathways between AD and PD. In contrast, only two of the top ten pathways identified by AD unique DEGs were also perturbed in PD, i.e., HIPPO Signalling and Sirtuin Signalling pathway. It is interesting that, of the top five perturbed pathways for the 2688 AD unique DEGs, neuroinflammation signalling pathway, complement system and NF-kB signalling were not perturbed in PD.

## Discussion

By integrating 126 brain samples from seven microarray gene expression datasets, we identified 1046 DEGs in PD. To our knowledge this is the largest meta-analysis study on microarray SN data about PD. Our approach allows inclusion of all the genes across all datasets included in this study. Only 267 out of the 1046 identified DEGs were included in all datasets. If only the common genes were used for meta-analysis, as applied in other previous gene expression meta-analysis about PD [23], we will have introduced many false negative results. This is because potentially interesting genes would not be identified when they are not common between studies. For instance, out of the top 30 identified DEGs, 14 would not have been identified, including *GBE1* [23] and *OPA1* [34] which have been associated with PD revealed in previous studies.

The gene *YWHAZ*, coding for the 14–3-3 zeta protein, was the top DEG and six 14–3-3 family proteins were important hubs in PPIN. Previously 14–3-3 proteins have been implicated in interactions with several proteins associated with PD including α-synuclein, Parkin and LRRK2 [35] and targeting 14–3-3 PPI using small molecules offers a promising strategy for PD and other neurodegenerative diseases [36]. 14–3-3 theta phosphorylation at S232 is observed in human PD brains to be pathogenic and contributes to the neurodegenerative process [37]. In Creutzfeldt-Jakob Disease (CJD) phosphorylation levels of 14–3-3 proteins have been used as a diagnostic biomarker clinically [35]. As we have found dysregulation of various 14–3-3 proteins in the post-mortem brain, further investigation into the potential of 14–3-3 protein dysregulation and phosphorylation levels as PD biomarkers in CSF and plasma is warranted.

Neuroinflammation is a typical part of the aging process [38] and it is accepted that the extent of neuroinflammation is greater in PD and AD patients [39]. We previously identified NF-kB as a TF in AD and LPS, a key regulator of reactive oxygen species (ROS) production, as a potential upstream regulator [7]. All of these can trigger a pro-inflammatory response. In our present study, inflammation pathways and upstream TFs that are pro-inflammatory are not perturbed in PD, suggesting a reduced importance of inflammation in the brain of patients with developed PD in comparison to AD. Degradation of dopamine is a major source of ROS in nigral tissue in PD brains, and late into PD development a lot of the dopamine producing cells are lost, potentially reducing inflammation levels [40]. Previously it has been shown that particular inflammation markers are not present in Parkinson’s disease dementia when compared to AD, suggesting that the neuroinflammatory mechanisms in PD and AD differ [41].

Although the DEGs between the two diseases were significantly overlapped, PD had a higher proportion that are also perturbed in AD. In addition, of the top five pathways perturbed in PD all were also perturbed in AD, however of the top 5 pathways perturbed in AD, only one was in PD. This suggests that processes underlying the two diseases are similar, however this it is more apparent with PD. Interestingly, the shared DEGs between PD and AD are almost always differentially expressed in the same up or down direction between diseases. This suggests that these genes could represent the crosstalk that is apparent between PD and AD. *MAPT* is one of four genes not differentially expressed in the same direction between the two diseases, being downregulated in AD and upregulated in PD. *MAPT* encodes the tau protein, and tau pathologies are important in both diseases [4, 42]. It has been shown that in three brain regions of AD patients there is a reduction in MAPT expression [43], however for PD it has been proposed that brain regions expressing greater levels of MAPT are more susceptible to tau mediated neurodegeneration [44]. This difference in *MAPT* and the role of tau pathology in both diseases warrants further investigation as these processes are not greatly understood.

Repressor element 1-silencing transcription factor (REST) has been implicated as an important regulator of neurons in the normal aging brain, closely correlating with cognitive longevity [45]. In AD and other dementias, REST is lost from the nucleus and is found with misfolded proteins in autophagosomes. *REST* was identified as an upstream regulator of down-regulated PD DEGs, as it has been in AD previously [7]. In cell models of PD, abnormal levels of the REST neuronal splice form REST4 have been implicated in pathology of PD [46]. It has been suggested that overexpression of α-synuclein affects the histone maker distribution on *REST* complex associated genes and results in repression of the *SNAP25* and *L1CAM* genes in both *Drosophila* and cell line models [47]. Reduction in these genes has been implicated in contributing to synaptic dysfunction in PD [47]. Here both genes have shown to be downregulated DEGs in PD supporting this mechanism underlying human PD pathogenesis.

The Sirtuin Signalling pathway was revealed to be perturbed in AD and PD and modulating their activities can alter the course of both diseases in both cell and animal models [48]. In PD SIRT1 and SIRT3 have protective effects against degeneration of SN neurons by neurotoxins, whereas activity of SIRT2 worsens the degeneration [48]. It is likely that SIRT1 and SIRT3 modulate homeostasis of mitochondria and anti-oxidative mechanisms, whereas activity of SIRT2 could result in adverse microtubule dynamics that disrupt clearance of toxic waste including Lewy bodies. In AD, the pan-sirtuin activator resveratrol has been shown to be safe, well-tolerated, and alter the trajectory of some biomarkers in a clinical trial [49]. Further research is needed to understand the therapeutic potential of sirtuins [48].

The SN was chosen as the brain region of interest in this study as neuron degeneration in this region is a hallmark of PD and it is the region with the most data for the meta-analysis [8]. We have excluded the SN microarray study GSE54282 from this meta-analysis due to low sample size and E-MEXP-1416 due to high variance in the data. There are also many studies using SN dopaminergic neurons, however including a number of these could lead the gene expression data to reflect these neuron types instead of the whole SN.

Although RNA-seq has demonstrated itself as a superior approach [50], there is not much data available for PD, although there is likely going to be further applications in the future. Microarrays are still very useful tools for measuring the gene expression and their power is further increased by using meta-analysis. Our microarray data has correlated gene expression values to the healthy SN RNA-seq data in the GTEx database [27], demonstrating that the microarray expression data used in this study has the similar quality to that of previous RNA-seq data (Additional file 1: Figure S2).

For PD there has been a limited application of RNA-seq to identify DEGs, in fact for the analysis of the SN only one RNA-seq study has been completed [51]. There are minimal similarities between the results of this RNA-seq analysis and our meta-analysis results. Only 70 of their 2961 identified DEGs are identified in our results, and only three of our top 30 DEGs (*SLC18A2, FGF13, AASDHPPT*) are identified in their results. However, pathways associated with oxidative phosphorylation, cardiac hypertrophy and the cytoskeleton were shared. A possible explanation for this is the very low power of the RNA-seq study, which only used three control and three PD samples and the fact that these samples were not age and gender matched. This is particularly important as age and gender are some of the largest risk factors for PD. The control samples had an average age of 87.3 (+/− 5.5) and were all females, and the PD samples had an average age of 79.0 (+/− 5.6) and only one sample was female.

A limitation of this study is that the SN is affected early in PD development, and by the time symptoms manifest much of the SN can be lost. This means our results reflect the perturbed genes and pathways present once the disease has been established, and not the changes that take place that lead to PD. To investigate early changes in disease more accessible tissues, such as blood and cerebrospinal fluid, would have to be investigated. Currently, there is no reliable way of diagnosing PD before it has had a substantial effect. As a result, investigating the perturbed pathways at this point in the disease would be difficult without development of effective early diagnosis biomarkers. Nonetheless identifying genes and pathways perturbed in the later stages of the disease can still help identify therapeutically important information and compare to similarly late stages of AD. A large limitation in this meta-analysis is the limited number of PD samples. As only 69 PD and 57 control samples are included in this study, the statistical power would be lower than that of our previous meta-analysis for AD which included 450 AD and 212 control samples [7]. This relatively low sample size could also introduce false positive and false negative DEGs and pathways, nevertheless, meta-analysis will outperform individual microarray studies. Moreover, our PPI networks would be best enriched by proteomics data of PD if such datasets are publicly available.

In conclusion, our meta-analysis strategy is the largest study of its type in PD SN tissue to date. We highlight REST as an important upstream regulator in PD and AD through the perturbation of Wnt signalling [45]. Our results reveal the importance of *YWHAZ* and 14–3-3 proteins in PD, through their down regulation, involvement in perturbed pathways and as hubs in PPIN. We demonstrate that PD and AD share significant number of DEGs that are differentially expressed in the same direction and perturbed pathways that indicate some novel shared pathogenesis between the two diseases. These insights suggest several new areas for mechanistic research into PD and cross-talk between AD and PD.

## Additional files

Additional file 1: **Table S1.** Information about each study used in our meta-analysis after removal of outlier samples. **Table S2.** Differentially expressed genes identified in our meta-analysis that have been identified as PD risk genes in a recent GWAS meta-analysis [33]. **Table S3.** IPA canonical pathway analysis for significant pathways identified using all PD DEGs, included with the information for pathways shared with those identified as significant using all AD DEGs. **Table S4.** IPA canonical pathway analysis for significant pathways identified using down-regulated PD DEGs. **Table S5.** IPA upstream regulator analysis for up and down regulated PD DEGs analysed separately. **Table S6.** Top 10 hubs found in the protein-protein interaction network (PPIN) analysis subnetwork created using the top 30 PD DEGs. **Table S7.** The direction of differential expression between the common DEGs found between AD and PD. **Figure S1.** Selecting filtering threshold for microarray data. The percentage of studies called absent in a mas5 present absent call for each probe was calculated, and threshold determined by minimizing Anderson-Darling normality tests and giving optimal Q-Q plot of the Z-scores after meta-analysis. The Q-Q plot for (A) 5%, (B) 10%, (C) 15%, (D) 20% and (E) 30% filtering. After 15% filtering A-D *p*-values were minimized (F) and the 15% Q-Q plot gave closest values to normality. A-D is Anderson-Darling normality test. **Figure S2.** RNAseq data vs. microarray gene expression data. Average absolute expression level of RNA-seq log2(TPM) of SN tissue from GTEx database plotted against RMA normalised and filtered intensity of microarray control and PD data used in this meta-analysis. The Pearson correlation coefficient between the control microarray data and healthy RNA-seq data (A) is 0.70 (*p*value < 2.2e-16) showing that the expression values of genes between microarray and RNA-seq are correlated and expression data distribution is similar. The Pearson correlation between the healthy RNA-seq and PD microarray data (B) is actually higher than between RNA-seq and control microarray at 0.73 (*p*value < 2.2e-16), when it would be expected to be lower due to some genes being differentially expressed. When using only DEGs, correlation between healthy RNA-seq and control microarray (C) and PD microarray (D) data this difference in correlation is minimised to 0.65 (*p*value < 2.2e-16) and 0.66 (*p*value < 2.2e-16) respectively, suggesting that the difference in correlation could be due to the larger sample size of the PD data. (DOCX 1024 kb)

Additional file 2: DEGs identified in this study. A full list of the 1046 DEGs identified in this meta-analysis in an Excel file. (XLSX 120 kb)

## Abbreviations

AD: Alzheimer’s disease
DEGs: Differentially expressed genes
FDR: False discovery rate
GTEx: Genotype-Tissue Expression
GWAS: Genome wide association study
IPA: Ingenuity® Pathway Analysis
PD: Parkinson’s disease
PPIN: Protein-protein interaction network
REST: Repressor element 1-silencing transcription factor
RMA: Robust multi-array average
RNA-seq: RNA sequencing
SN: Substantia nigra
TF: Transcription factor
TPM: Transcripts per kilobase million
