# Supplementary material for: Gene expression meta-analysis of Parkinson’s disease and its relationship with Alzheimer’s disease
Source: Mol Brain. 2019 Feb 28;12:16. doi: 10.1186/s13041-019-0436-5 (PMC6396547; doi:10.1186/s13041-019-0436-5)
Supplement: Supplementary file 1 — Table S1. Information about each study used in our meta-analysis after removal of outlier samples. Table S2. Differentially expressed genes identified in our meta-analysis that have been identified as PD risk genes in a recent GWAS meta-analysis [33]. Table S3. IPA canonical pathway analysis for significant pathways identified using all PD DEGs, included with the information for pathways shared with those identified as significant using all AD DEGs. Table S4. IPA canonical pathway analysis for significant pathways identified using down-regulated PD DEGs. Table S5. IPA upstream regulator analysis for up and down regulated PD DEGs analysed separately. Table S6. Top 10 hubs found in the protein-protein interaction network (PPIN) analysis subnetwork created using the top 30 PD DEGs. Table S7. The direction of differential expression between the common DEGs found between AD and PD. Figure S1. Selecting filtering threshold for microarray data. The percentage of studies called absent in a mas5 present absent call for each probe was calculated, and threshold determined by minimizing Anderson-Darling normality tests and giving optimal Q-Q plot of the Z-scores after meta-analysis. The Q-Q plot for (A) 5%, (B) 10%, (C) 15%, (D) 20% and (E) 30% filtering. After 15% filtering A-D p-values were minimized (F) and the 15% Q-Q plot gave closest values to normality. A-D is Anderson-Darling normality test. Figure S2. RNAseq data vs. microarray gene expression data. Average absolute expression level of RNA-seq log2(TPM) of SN tissue from GTEx database plotted against RMA normalised and filtered intensity of microarray control and PD data used in this meta-analysis. The Pearson correlation coefficient between the control microarray data and healthy RNA-seq data (A) is 0.70 (pvalue < 2.2e-16) showing that the expression values of genes between microarray and RNA-seq are correlated and expression data distribution is similar. The Pearson correlation between the healthy RNA-seq and PD micr [file 13041_2019_436_MOESM1_ESM.docx]

**Gene expression meta-analysis of Parkinson’s disease and its relationship with Alzheimer’s disease**

Additional file 1

| **Table S1: Information about each study used in our meta-analysis** | | | | | | | |
| --- | --- | --- | --- | --- | --- | --- | --- |
| **GEO Accession number** | **Platform name** | **Platform ID** |  | **Male** | **Female** | **All** | **Age range (average)** |
| GSE7621 | Affymetrix Human Genome U133 Plus 2.0 Array | GPL570 | PD | 13 | 3 | 16 | N/A |
|  |  |  | Control | 4 | 5 | 9 | N/A |
| GSE20141 | Affymetrix Human Genome U133 Plus 2.0 Array | GPL570 | PD | N/A | N/A | 9 | N/A |
|  |  |  | Control | N/A | N/A | 6 | N/A |
| GSE8397 | Affymetrix Human Genome U133A Array | GPL96 | PD | 9 | 6 | 15 | 68-89 (80) |
|  |  |  | Control | 5 | 1 | 6 | 46-81 (68.2) |
| GSE20292 | Affymetrix Human Genome U133A Array | GPL96 | PD | 6 | 5 | 11 | 67-84 (75.5) |
|  |  |  | Control | 13 | 5 | 18 | 41-94 (66.8) |
| GSE20163 | Affymetrix Human Genome U133A Array | GPL96 | PD | N/A | N/A | 8 | N/A |
|  |  |  | Control | N/A | N/A | 9 | N/A |
| GSE20164 | Affymetrix Human Genome U133A Array | GPL96 | PD | N/A | N/A | 6 | N/A |
|  |  |  | Control | N/A | N/A | 3 | N/A |
| GSE20333 | Affymetrix Human HG-Focus Target Array | GPL201 | PD | 1 | 3 | 4 | 70-87 (77.3) |
|  |  |  | Control | 5 | 1 | 6 | 68-88 (79) |

Table S1. Information about each study used in our meta-analysis after removal of outlier samples.

| **Table S2: DEGs that have been previously identified as PD risk genes by GWAS** | | | | |
| --- | --- | --- | --- | --- |
| **Gene name** | **Entrez ID** | **Average FC** | **metaZscore** | **FDR corrected Pval** |
| SNCA | 6622 | 0.57 | -6.00 | 1.03E-05 |
| ANK2 | 287 | 0.61 | -4.21 | 2.33E-03 |
| ALAS1 | 211 | 0.76 | -3.52 | 1.12E-02 |
| SH3GL2 | 6456 | 0.64 | -3.46 | 1.31E-02 |
| DLG2 | 1740 | 0.79 | -3.34 | 1.68E-02 |
| SCN3A | 6328 | 0.56 | -3.30 | 1.79E-02 |
| MAPT | 4137 | 1.23 | 3.15 | 2.45E-02 |
| ATP6V0A1 | 535 | 0.85 | -3.03 | 3.15E-02 |
| VPS13C | 54832 | 1.17 | 2.85 | 4.61E-02 |

Table S2: Differentially expressed genes identified in our meta-analysis that have been identified as PD risk genes in a recent GWAS meta-analysis [33].

| *Table S3* | | | | | | | |
| --- | --- | --- | --- | --- | --- | --- | --- |
| *Ingenuity Canonical Pathways* | *DEGs* | *Genes in Pathway* | *Ratio* | *adjPval* | *AD DEGs* | *AD Ratio* | *AD adjPval* |
| Breast Cancer Regulation by Stathmin1 | 33 | 204 | 0.162 | 2.40E-06 | 47 | 0.23 | 8.51E-03 |
| Sirtuin Signaling Pathway | 40 | 284 | 0.141 | 2.40E-06 | 70 | 0.247 | 3.39E-04 |
| 14-3-3-mediated Signaling | 24 | 130 | 0.185 | 9.55E-06 | 34 | 0.262 | 5.13E-03 |
| Phagosome Maturation | 23 | 138 | 0.167 | 9.12E-05 | 34 | 0.246 | 1.15E-02 |
| Remodeling of Epithelial Adherens Junctions | 15 | 67 | 0.227 | 1.12E-04 |  |  |  |
| Mitochondrial Dysfunction | 24 | 166 | 0.145 | 4.27E-04 | 41 | 0.248 | 4.79E-03 |
| Oxidative Phosphorylation | 18 | 105 | 0.173 | 4.68E-04 |  |  |  |
| CDK5 Signaling | 17 | 99 | 0.173 | 7.24E-04 | 27 | 0.276 | 7.08E-03 |
| Huntington's Disease Signaling | 30 | 248 | 0.121 | 7.59E-04 |  |  |  |
| B Cell Receptor Signaling | 25 | 188 | 0.133 | 7.59E-04 | 50 | 0.266 | 6.92E-04 |
| Epithelial Adherens Junction Signaling | 21 | 143 | 0.147 | 7.59E-04 |  |  |  |
| Gap Junction Signaling | 24 | 191 | 0.126 | 2.51E-03 |  |  |  |
| Germ Cell-Sertoli Cell Junction Signaling | 22 | 170 | 0.130 | 2.69E-03 | 41 | 0.243 | 7.08E-03 |
| Axonal Guidance Signaling | 43 | 447 | 0.096 | 2.95E-03 | 94 | 0.211 | 2.09E-03 |
| Sertoli Cell-Sertoli Cell Junction Signaling | 22 | 174 | 0.127 | 3.31E-03 | 38 | 0.22 | 3.72E-02 |
| Rac Signaling | 17 | 116 | 0.147 | 3.31E-03 | 29 | 0.25 | 1.70E-02 |
| Synaptic Long Term Potentiation | 17 | 119 | 0.143 | 4.17E-03 | 29 | 0.244 | 2.34E-02 |
| AMPK Signaling | 25 | 216 | 0.116 | 4.17E-03 |  |  |  |
| Glycolysis I | 7 | 24 | 0.292 | 4.27E-03 |  |  |  |
| PI3K/AKT Signaling | 17 | 124 | 0.138 | 5.50E-03 | 34 | 0.276 | 2.09E-03 |
| ERK/MAPK Signaling | 23 | 199 | 0.116 | 7.08E-03 | 51 | 0.256 | 9.55E-04 |
| Iron homeostasis signaling pathway | 17 | 127 | 0.134 | 7.41E-03 |  |  |  |
| Clathrin-mediated Endocytosis Signaling | 23 | 206 | 0.112 | 1.05E-02 |  |  |  |
| Gαi Signaling | 16 | 121 | 0.133 | 1.05E-02 |  |  |  |
| Signaling by Rho Family GTPases | 26 | 250 | 0.104 | 1.38E-02 | 61 | 0.243 | 9.55E-04 |
| Neuropathic Pain Signaling In Dorsal Horn Neurons | 15 | 114 | 0.132 | 1.62E-02 |  |  |  |
| G Protein Signaling Mediated by Tubby | 7 | 31 | 0.226 | 1.62E-02 |  |  |  |
| Role of NFAT in Cardiac Hypertrophy | 23 | 217 | 0.106 | 1.62E-02 | 47 | 0.218 | 2.19E-02 |
| Reelin Signaling in Neurons | 13 | 93 | 0.141 | 1.62E-02 |  |  |  |
| Amyloid Processing | 9 | 50 | 0.180 | 1.62E-02 |  |  |  |
| Cardiac β-adrenergic Signaling | 17 | 141 | 0.121 | 1.62E-02 |  |  |  |
| TCA Cycle II (Eukaryotic) | 6 | 24 | 0.250 | 1.82E-02 |  |  |  |
| p70S6K Signaling | 16 | 132 | 0.122 | 1.95E-02 |  |  |  |
| PTEN Signaling | 15 | 120 | 0.126 | 1.95E-02 | 31 | 0.261 | 7.94E-03 |
| Sumoylation Pathway | 13 | 97 | 0.135 | 2.00E-02 | 30 | 0.312 | 9.33E-04 |
| Protein Ubiquitination Pathway | 26 | 264 | 0.099 | 2.00E-02 |  |  |  |
| Gluconeogenesis I | 6 | 25 | 0.240 | 2.00E-02 |  |  |  |
| STAT3 Pathway | 13 | 98 | 0.134 | 2.04E-02 | 20 | 0.27 | 2.63E-02 |
| HIPPO signaling | 12 | 86 | 0.140 | 2.09E-02 | 32 | 0.372 | 4.17E-05 |
| fMLP Signaling in Neutrophils | 15 | 122 | 0.123 | 2.14E-02 | 33 | 0.27 | 3.47E-03 |
| Dopamine-DARPP32 Feedback in cAMP Signaling | 18 | 161 | 0.112 | 2.14E-02 |  |  |  |
| D-myo-inositol (1,4,5)-trisphosphate Degradation | 5 | 18 | 0.278 | 2.14E-02 |  |  |  |
| Dopamine Receptor Signaling | 11 | 76 | 0.145 | 2.14E-02 |  |  |  |
| GNRH Signaling | 18 | 163 | 0.111 | 2.29E-02 | 38 | 0.235 | 1.45E-02 |
| Insulin Receptor Signaling | 16 | 137 | 0.117 | 2.34E-02 |  |  |  |
| Cardiac Hypertrophy Signaling | 23 | 233 | 0.099 | 2.63E-02 | 51 | 0.219 | 1.45E-02 |
| Cyclins and Cell Cycle Regulation | 11 | 80 | 0.138 | 3.02E-02 | 20 | 0.253 | 4.79E-02 |
| IGF-1 Signaling | 13 | 106 | 0.123 | 3.55E-02 |  |  |  |
| Protein Kinase A Signaling | 33 | 386 | 0.086 | 3.72E-02 | 82 | 0.214 | 3.24E-03 |
| Gαq Signaling | 17 | 159 | 0.107 | 3.89E-02 | 42 | 0.264 | 1.58E-03 |
| Aspartate Degradation II | 3 | 7 | 0.429 | 3.98E-02 | 5 | 0.714 | 9.55E-03 |
| Renin-Angiotensin Signaling | 14 | 121 | 0.116 | 3.98E-02 |  |  |  |
| ATM Signaling | 12 | 97 | 0.124 | 4.27E-02 |  |  |  |
| BMP signaling pathway | 10 | 75 | 0.135 | 4.47E-02 |  |  |  |

Table S3: IPA canonical pathway analysis for significant pathways identified using all PD DEGs, included with the information for pathways shared with those identified as significant using all AD DEGs.

| *Table S4:* *IPA canonical pathway analysis for significant pathways identified using down-regulated PD DEGs.* | | | | |
| --- | --- | --- | --- | --- |
| Ingenuity Canonical Pathways | DEGs | Genes in Pathway | Ratio | Adj. Pval |
| Breast Cancer Regulation by Stathmin1 | 32 | 204 | 0.157 | 1.86E-09 |
| Phagosome Maturation | 23 | 138 | 0.167 | 2.19E-07 |
| Sirtuin Signaling Pathway | 34 | 284 | 0.120 | 2.19E-07 |
| Mitochondrial Dysfunction | 24 | 166 | 0.145 | 9.55E-07 |
| 14-3-3-mediated Signaling | 21 | 130 | 0.162 | 9.55E-07 |
| Remodeling of Epithelial Adherens Junctions | 15 | 67 | 0.227 | 9.55E-07 |
| Oxidative Phosphorylation | 18 | 105 | 0.173 | 2.82E-06 |
| Axonal Guidance Signaling | 39 | 447 | 0.087 | 3.39E-05 |
| Gap Junction Signaling | 22 | 192 | 0.115 | 1.17E-04 |
| CDK5 Signaling | 15 | 99 | 0.153 | 1.32E-04 |
| Huntington's Disease Signaling | 25 | 248 | 0.101 | 2.14E-04 |
| Sertoli Cell-Sertoli Cell Junction Signaling | 20 | 173 | 0.116 | 2.34E-04 |
| Germ Cell-Sertoli Cell Junction Signaling | 19 | 170 | 0.112 | 5.13E-04 |
| Cardiac β-adrenergic Signaling | 17 | 141 | 0.121 | 5.13E-04 |
| Glycolysis I | 7 | 24 | 0.292 | 5.37E-04 |
| Epithelial Adherens Junction Signaling | 17 | 143 | 0.119 | 6.03E-04 |
| Synaptic Long Term Potentiation | 15 | 120 | 0.126 | 8.51E-04 |
| PI3K/AKT Signaling | 15 | 123 | 0.122 | 1.17E-03 |
| Iron homeostasis signaling pathway | 15 | 128 | 0.118 | 1.62E-03 |
| Neuropathic Pain Signaling In Dorsal Horn Neurons | 14 | 114 | 0.123 | 1.74E-03 |
| Dopamine-DARPP32 Feedback in cAMP Signaling | 17 | 161 | 0.106 | 1.91E-03 |
| Rac Signaling | 14 | 116 | 0.121 | 1.95E-03 |
| Dopamine Receptor Signaling | 11 | 76 | 0.145 | 1.99E-03 |
| Role of NFAT in Cardiac Hypertrophy | 20 | 216 | 0.093 | 2.88E-03 |
| Signaling by Rho Family GTPases | 22 | 252 | 0.088 | 2.95E-03 |
| TCA Cycle II (Eukaryotic) | 6 | 24 | 0.250 | 3.24E-03 |
| Protein Kinase A Signaling | 29 | 386 | 0.075 | 3.80E-03 |
| Gluconeogenesis I | 6 | 25 | 0.240 | 3.80E-03 |
| HIPPO signaling | 11 | 86 | 0.128 | 4.79E-03 |
| GNRH Signaling | 16 | 162 | 0.099 | 5.01E-03 |
| p70S6K Signaling | 14 | 131 | 0.107 | 5.01E-03 |
| AMPK Signaling | 19 | 215 | 0.088 | 5.62E-03 |
| ERK/MAPK Signaling | 18 | 199 | 0.091 | 5.89E-03 |
| Amyloid Processing | 8 | 50 | 0.160 | 5.89E-03 |
| Gαi Signaling | 13 | 121 | 0.108 | 6.17E-03 |
| Opioid Signaling Pathway | 20 | 237 | 0.084 | 6.46E-03 |
| fMLP Signaling in Neutrophils | 13 | 122 | 0.107 | 6.92E-03 |
| Clathrin-mediated Endocytosis Signaling | 18 | 206 | 0.087 | 7.59E-03 |
| CREB Signaling in Neurons | 18 | 211 | 0.086 | 8.91E-03 |
| Gαq Signaling | 15 | 160 | 0.094 | 8.91E-03 |
| Protein Ubiquitination Pathway | 21 | 265 | 0.080 | 8.91E-03 |
| RhoGDI Signaling | 16 | 177 | 0.091 | 8.91E-03 |
| Melatonin Signaling | 9 | 70 | 0.129 | 1.10E-02 |
| Calcium Signaling | 17 | 198 | 0.086 | 1.12E-02 |
| α-Adrenergic Signaling | 10 | 85 | 0.118 | 1.12E-02 |
| Synaptic Long Term Depression | 15 | 168 | 0.089 | 1.38E-02 |
| Actin Cytoskeleton Signaling | 18 | 222 | 0.081 | 1.38E-02 |
| BMP signaling pathway | 9 | 74 | 0.122 | 1.41E-02 |
| Aspartate Degradation II | 3 | 7 | 0.429 | 1.41E-02 |
| B Cell Receptor Signaling | 16 | 189 | 0.085 | 1.48E-02 |
| Insulin Receptor Signaling | 13 | 137 | 0.095 | 1.48E-02 |
| Regulation of eIF4 and p70S6K Signaling | 14 | 155 | 0.091 | 1.48E-02 |
| IGF-1 Signaling | 11 | 106 | 0.104 | 1.55E-02 |
| Reelin Signaling in Neurons | 10 | 92 | 0.109 | 1.70E-02 |
| Fcγ Receptor-mediated Phagocytosis in Macrophages and Monocytes | 10 | 93 | 0.108 | 1.82E-02 |
| Parkinson's Signaling | 4 | 16 | 0.250 | 1.86E-02 |
| Pyridoxal 5'-phosphate Salvage Pathway | 8 | 64 | 0.125 | 1.86E-02 |
| CCR3 Signaling in Eosinophils | 12 | 127 | 0.095 | 1.86E-02 |
| Cardiac Hypertrophy Signaling | 18 | 232 | 0.078 | 1.86E-02 |
| Phototransduction Pathway | 7 | 52 | 0.137 | 1.91E-02 |
| Salvage Pathways of Pyrimidine Ribonucleotides | 10 | 96 | 0.105 | 1.91E-02 |
| D-myo-inositol (1,4,5)-Trisphosphate Biosynthesis | 5 | 28 | 0.185 | 2.00E-02 |
| Cdc42 Signaling | 12 | 130 | 0.093 | 2.08E-02 |
| Tight Junction Signaling | 14 | 167 | 0.084 | 2.40E-02 |
| Chemokine Signaling | 8 | 68 | 0.118 | 2.40E-02 |
| Aldosterone Signaling in Epithelial Cells | 14 | 168 | 0.084 | 2.45E-02 |
| D-myo-inositol (1,4,5)-trisphosphate Degradation | 4 | 19 | 0.222 | 2.45E-02 |
| P2Y Purigenic Receptor Signaling Pathway | 12 | 134 | 0.090 | 2.45E-02 |
| PAK Signaling | 10 | 100 | 0.100 | 2.45E-02 |
| Role of CHK Proteins in Cell Cycle Checkpoint Control | 7 | 57 | 0.123 | 3.09E-02 |
| G Protein Signaling Mediated by Tubby | 5 | 32 | 0.161 | 3.16E-02 |
| Renin-Angiotensin Signaling | 11 | 122 | 0.091 | 3.16E-02 |
| Inhibition of Angiogenesis by TSP1 | 5 | 33 | 0.156 | 3.63E-02 |
| Xenobiotic Metabolism Signaling | 19 | 273 | 0.070 | 3.80E-02 |
| G-Protein Coupled Receptor Signaling | 19 | 275 | 0.069 | 3.98E-02 |
| GDNF Family Ligand-Receptor Interactions | 8 | 77 | 0.105 | 3.98E-02 |
| IL-1 Signaling | 9 | 93 | 0.098 | 3.98E-02 |
| Ceramide Signaling | 9 | 93 | 0.097 | 4.17E-02 |
| Arsenate Detoxification I (Glutaredoxin) | 2 | 4 | 0.500 | 4.37E-02 |
| CXCR4 Signaling | 13 | 164 | 0.079 | 4.37E-02 |
| Mevalonate Pathway I | 3 | 12 | 0.250 | 4.68E-02 |

Table S4: IPA canonical pathway analysis for significant pathways identified using down-regulated PD DEGs.

| *Table S5: IPA upstream regulator analysis for up and down regulated PD DEGs analysed separately.* | | | |
| --- | --- | --- | --- |
| Upstream Regulator | Molecule type | p-value | Number of target molecules |
| **Upstream regulators for Down-regulated DEGs** | | | |
| Lh | complex | 1.21E-08 | 27 |
| FSH | complex | 7.25E-07 | 28 |
| HSP90B1 | other | 8.38E-05 | 7 |
| CUL4B | other | 2.03E-04 | 5 |
| SBDS | other | 2.67E-04 | 11 |
| REST | transcription regulator | 2.91E-04 | 6 |
| SUZ12 | enzyme | 4.01E-04 | 11 |
| LONP1 | peptidase | 6.46E-04 | 9 |
| MMP12 | peptidase | 1.51E-03 | 7 |
| INHBA | growth factor | 3.64E-03 | 10 |
| NMNAT1 | enzyme | 4.84E-03 | 3 |
| PRKAR1A | kinase | 4.84E-03 | 3 |
| RBM5 | other | 5.10E-03 | 6 |
| IL15 | cytokine | 5.16E-03 | 10 |
| HNRNPA2B1 | other | 5.42E-03 | 12 |
| CCND1 | transcription regulator | 5.87E-03 | 16 |
| TP53 | transcription regulator | 7.91E-03 | 37 |
| **Upstream regulators for Up-regulated DEGs** | | | |
| HSF1 | transcription regulator | 1.57E-04 | 8 |
| TGFBR2 | kinase | 5.16E-04 | 6 |
| miR-346 (and other miRNAs w/seed GUCUGCC) | mature microrna | 6.73E-04 | 2 |
| TP73 | transcription regulator | 8.09E-04 | 9 |
| SP4 | transcription regulator | 1.09E-03 | 3 |
| MTOR | kinase | 1.20E-03 | 5 |
| NPAT | transcription regulator | 1.33E-03 | 2 |
| AREG | growth factor | 2.60E-03 | 5 |
| COL18A1 | other | 3.18E-03 | 5 |
| ZBTB10 | other | 3.27E-03 | 2 |
| MYC | transcription regulator | 4.07E-03 | 10 |
| miR-22-3p (miRNAs w/seed AGCUGCC) | mature microrna | 4.23E-03 | 3 |
| CD24 | other | 4.50E-03 | 6 |
| ZNF652 | other | 4.53E-03 | 2 |
| CCND1 | transcription regulator | 5.50E-03 | 9 |
| GATA6 | transcription regulator | 5.86E-03 | 4 |
| Cdk | group | 6.11E-03 | 3 |
| SAFB | other | 7.26E-03 | 4 |
| mir-122 | microrna | 8.41E-03 | 5 |
| miR-155-5p (miRNAs w/seed UAAUGCU) | mature microrna | 8.42E-03 | 3 |
| E2F1 | transcription regulator | 8.87E-03 | 8 |
| KITLG | growth factor | 9.28E-03 | 3 |
| CASP8 | peptidase | 9.42E-03 | 2 |
| DDIT3 | transcription regulator | 9.42E-03 | 2 |
| CBL | transcription regulator | 9.42E-03 | 2 |

Table S5: IPA upstream regulator analysis for up and down regulated PD DEGs analyzed separately.

| **Table S6:** **Top 10 hubs found in the PPIN subnetwork created using the top 30 PD DEGs** | |
| --- | --- |
| **Gene name** | **Number of First neighbour nodes** |
| YWHAZ | 122 |
| YWHAB | 62 |
| YWHAG | 62 |
| YWHAE | 42 |
| YWHAQ | 39 |
| SNCA | 38 |
| YWHAH | 32 |
| TP53 | 25 |
| AKT1 | 22 |
| ABL1 | 19 |

Table S6: Top 10 hubs found in the protein-protein interaction network (PPIN) analysis subnetwork created using the top 30 PD DEGs.

| **Table S7: The direction of differential expression between the common DEGs of AD and PD.** | | | |
| --- | --- | --- | --- |
|  | **PD upregulated** | **PD downregulated** | **Total** |
| AD upregulated | 114 | 3 | 117 |
| AD downregulated | 1 | 318 | 319 |
| Total | 115 | 321 | 436 |

Table S7: The direction of differential expression between the common DEGs found between AD and PD.


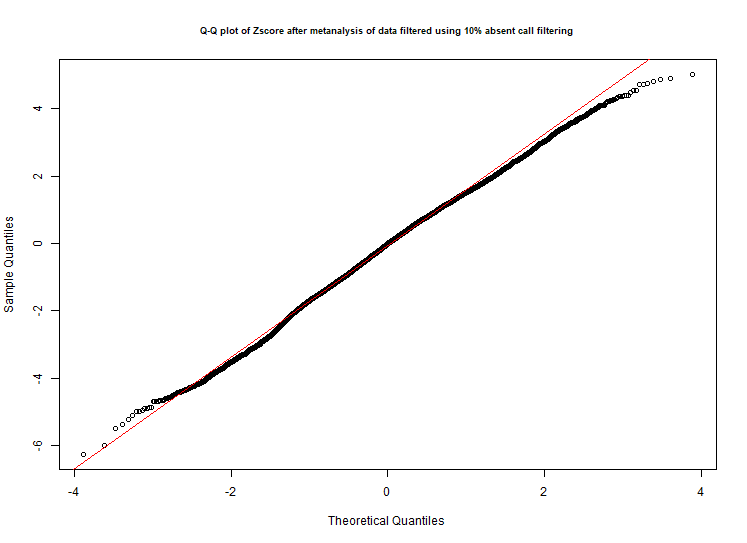

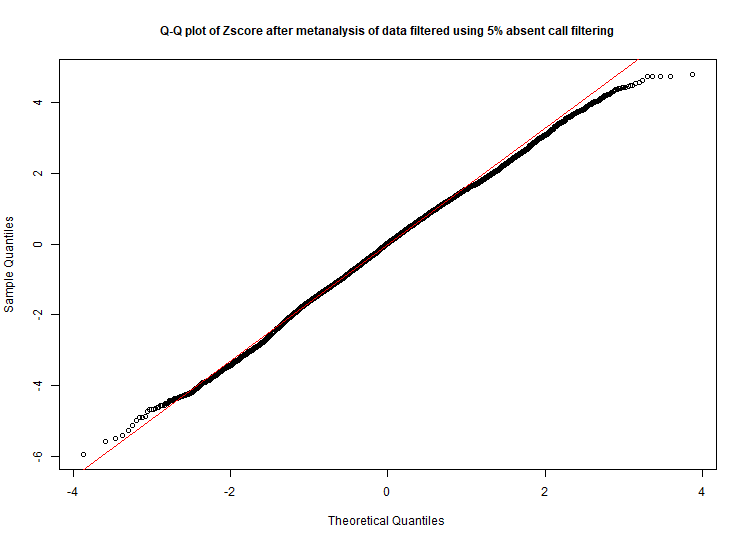

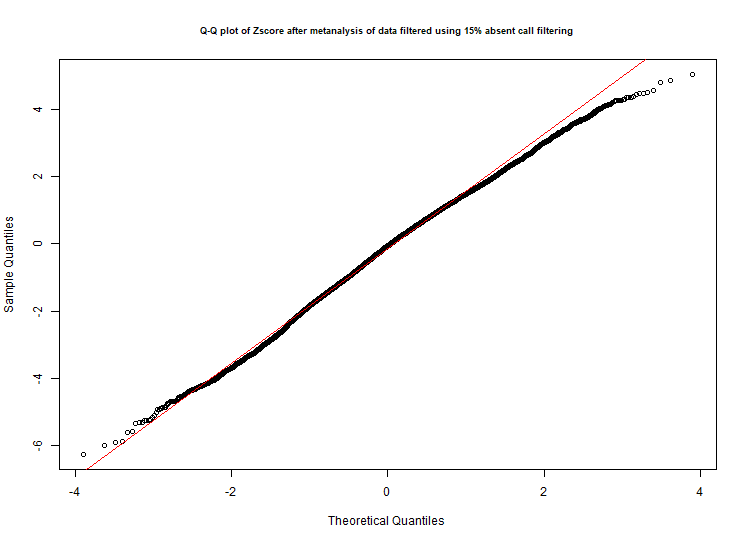

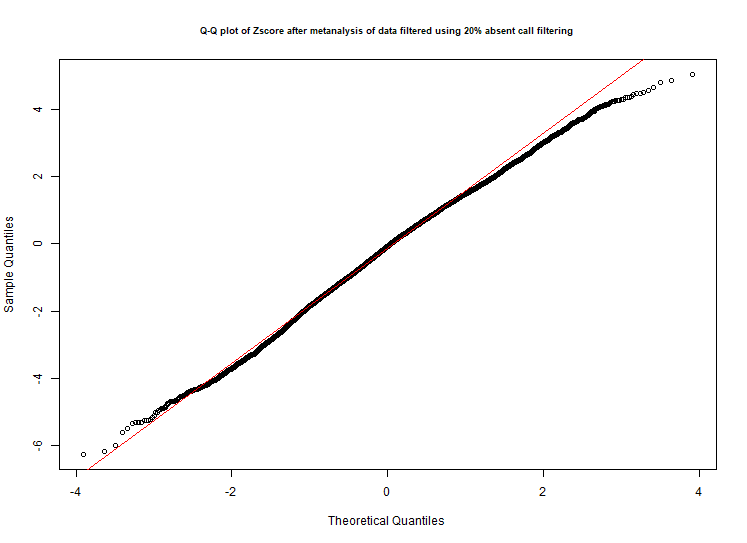

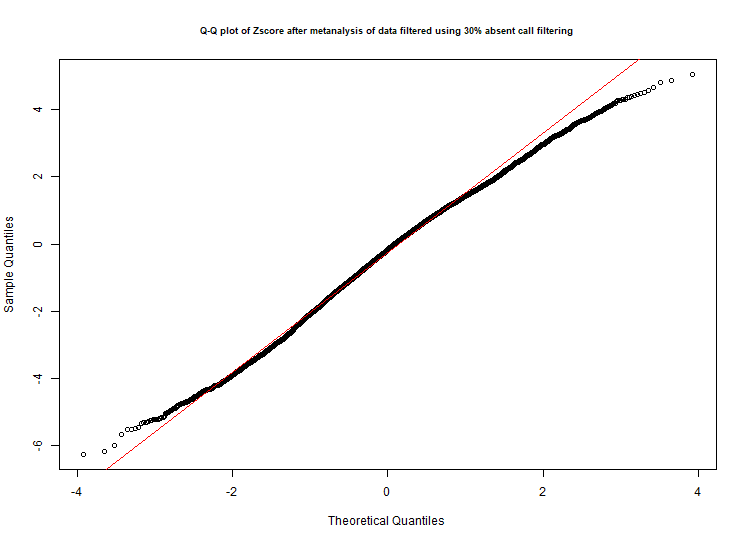


A

B

D

C

E

F


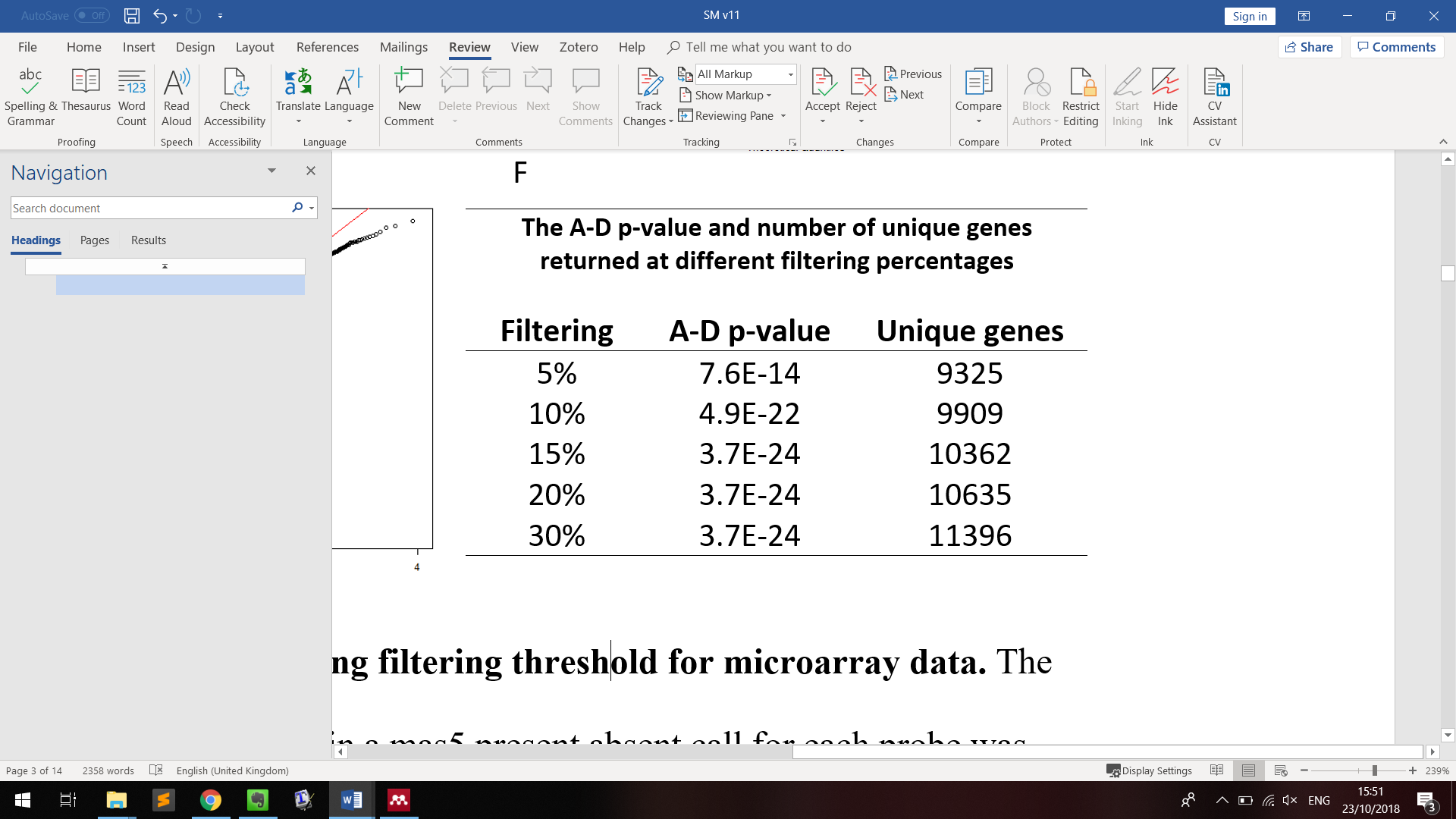


**Figure S1: Selecting filtering threshold for microarray data.** The percentage of studies called absent in a mas5 present absent call for each probe was calculated, and threshold determined by minimizing Anderson-Darling normality tests and giving optimal Q-Q plot of the Z-scores after meta-analysis. The Q-Q plot for (A) 5%, (B) 10%, (C) 15%, (D) 20% and (E) 30% filtering. After 15% filtering A-D p-values were minimized (F) and the 15% Q-Q plot gave closest values to normality. A-D is Anderson-Darling normality test.


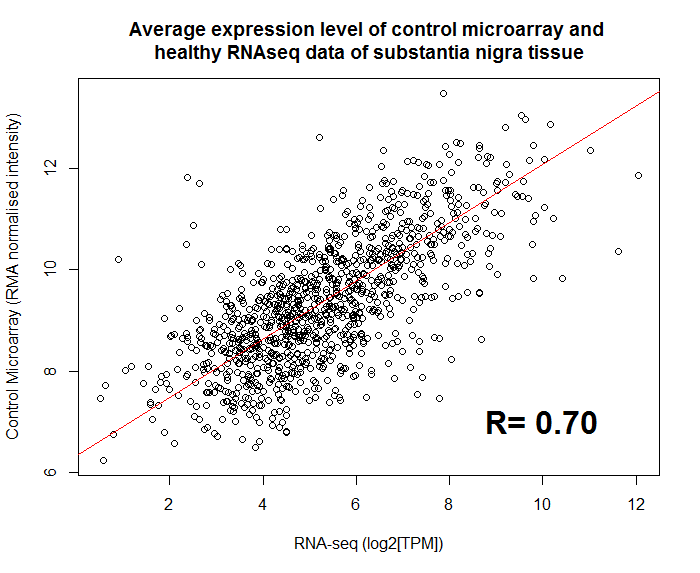


A


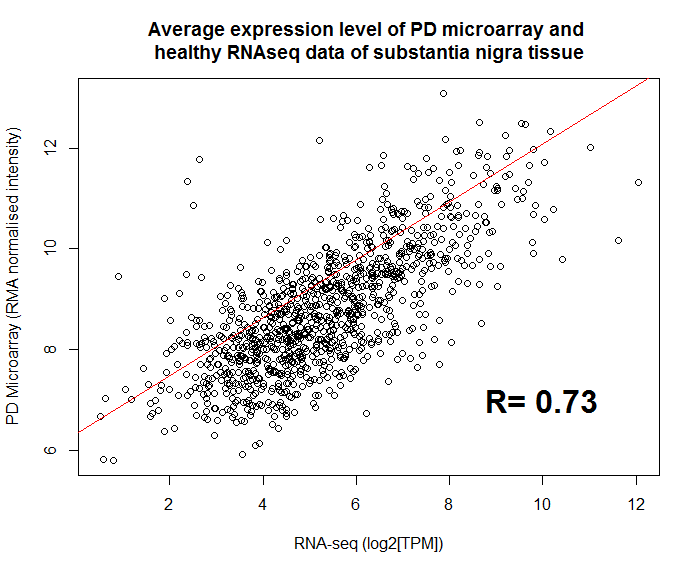

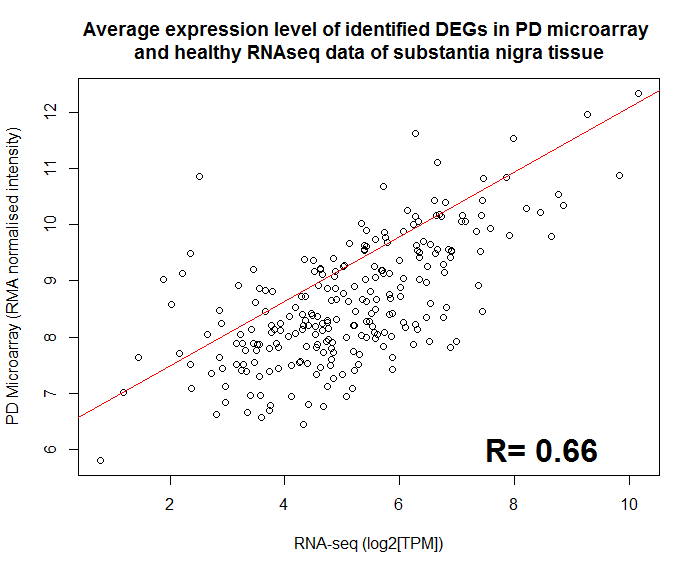


D

B


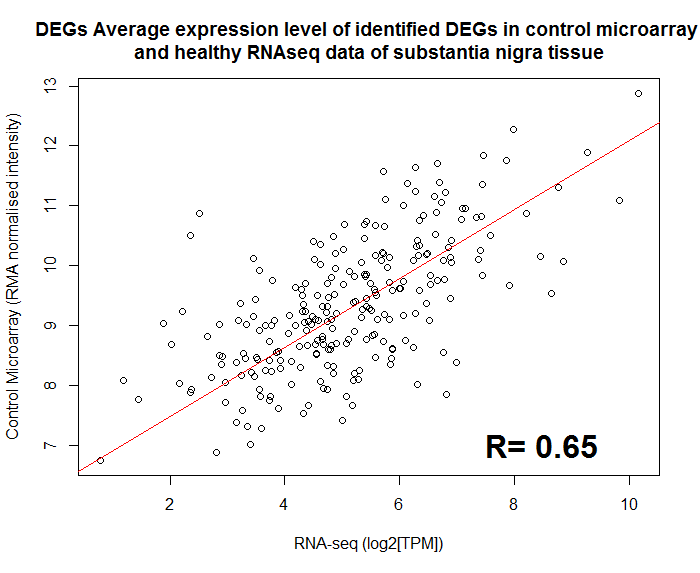


C

**Figure S2. RNA-seq vs microarray.** Average absolute expression level of RNA-seq log2(TPM) of SN tissue from GTEx database plotted against RMA normalised and filtered intensity of microarray control and PD data used in this meta-analysis**.** The Pearson correlation coefficient between the control microarray data and healthy RNA-seq data (A) is 0.70 (pvalue < 2.2e-16) showing that the expression values of genes between microarray and RNA-seq are correlated and expression data distribution is similar. The Pearson correlation between the healthy RNA-seq and PD microarray data (B) is actually higher than between RNA-seq and control microarray at 0.73 (pvalue < 2.2e-16), when it would be expected to be lower due to some genes being differentially expressed. When using only DEGs, correlation between healthy RNA-seq and control microarray (C) and PD microarray (D) data this difference in correlation is minimised to 0.65 (pvalue < 2.2e-16) and 0.66 (pvalue < 2.2e-16) respectively, suggesting that the difference in correlation could be due to the larger sample size of the PD data.
